# Deriving a light use efficiency estimation algorithm using *in situ* hyperspectral and eddy covariance measurements for a maize canopy in Northeast China

**DOI:** 10.1002/ece3.3051

**Published:** 2017-05-23

**Authors:** Feng Zhang, Guangsheng Zhou

**Affiliations:** ^1^ Chinese Academy of Meteorological SciencesBeijingChina; ^2^ State Key Laboratory of Vegetation and Environmental ChangeInstitute of BotanyChinese Academy of SciencesBeijingChina

**Keywords:** canopy chlorophyll content, eddy covariance, hyperspectral remote sensing, light use efficiency, spectral vegetation indices

## Abstract

We estimated the light use efficiency (*LUE*) via vegetation canopy chlorophyll content (*CCC*_canopy_) based on *in situ* measurements of spectral reflectance, biophysical characteristics, ecosystem CO_2_ fluxes and micrometeorological factors over a maize canopy in Northeast China. The results showed that among the common chlorophyll‐related vegetation indices (VIs), *CCC*_canopy_ had the most obviously exponential relationships with the red edge position (REP) (*R* ^2^ = .97, *p *<* *.001) and normalized difference vegetation index (NDVI) (*R* ^2^ = .91, *p *<* *.001). In a comparison of the indicating performances of NDVI, ratio vegetation index (RVI), wide dynamic range vegetation index (WDRVI), and 2‐band enhanced vegetation index (EVI2) when estimating *CCC*_canopy_ using all of the possible combinations of two separate wavelengths in the range 400−1300 nm, EVI2 [1214, 1259] and EVI2 [726, 1248] were better indicators, with *R* ^2^ values of .92 and .90 (*p *<* *.001). Remotely monitoring *LUE* through estimating *CCC*_canopy_ derived from field spectrometry data provided accurate prediction of midday gross primary productivity (*GPP*) in a rainfed maize agro‐ecosystem (*R* ^2^ = .95, *p *<* *.001). This study provides a new paradigm for monitoring vegetation *GPP* based on the combination of *LUE* models with plant physiological properties.

## 1 INTRODUCTION

The accurate assessment of vegetation gross primary productivity (*GPP*) is of great importance for regional and global studies of terrestrial ecosystem carbon budgets (Gitelson et al., 2006; Peng & Gitelson, 2011; Wu, Niu, & Gao, 2012), and it also plays a significant role in dynamic responses of terrestrial ecosystem carbon cycling to global climate change (Fang, Yu, & Qi, 2015; Fang & Zhang, 2013; Shen & Fang, 2014). The eddy covariance (*EC*) technique provides long‐term continuous and frequent observations of CO_2_ flux at the ecosystem level (e.g., Baldocchi, 2003). Remote sensing techniques conduct consistent and systematic monitoring of vegetation structure and function at the regional and site levels (Ide, Nakaji, & Oguma, 2010; Lawley et al., 2016; Running, Thornton, Nemani, & Glassy, 2000). How to effectively relate CO_2_ flux observations with remote sensing techniques at the site level and ultimately to implement repetitive observations of CO_2_ flux over extensive spatial areas are becoming critical challenges for assessing global carbon budgets and monitoring ecosystem dynamical processes. The key for addressing these questions lies in the development of remote sensing‐based ecosystem process models at broad spatial scales that can be effectively and quantitatively parameterized and validated by CO_2_ fluxes at site level.

Currently, the accurate estimations of the fraction of absorbed photosynthetically active radiation (*fAPAR*) and the light use efficiency (*LUE*) are two large sources of model uncertainties for *LUE* models (Inoue, Peñuelas, Miyata, & Mano, 2008; Peng & Gitelson, 2011). On the one hand, studies showed that the sensitivity of the normalized difference vegetation index (NDVI) to variations in *fAPAR* usually decreases when *fAPAR* exceeds 0.7 for moderate‐to‐high vegetation density (Viña & Gitelson, 2005), moreover, the relationship of NDVI‐*fAPAR* was also influenced by plant phenology (e.g., Jenkins et al., 2007; Running et al., 2000). On the other hand, studies have demonstrated that *LUE* was not a prescribed constant during the whole growing season (e.g., Jarvis & Leverenz, 1983) and was not only related to the absorbed photosynthetically active radiation (*APAR*) by green vegetation but also affected by the soil water content (*SWC*), nutrient conditions, ratio of direct to diffuse radiation, canopy age, and site history (Alton, North, & Los, 2007; DeLucia, Drake, Thomas, & Gonzalez‐Meler, 2007). Thus, studies on how to effectively improve the accuracy of remote estimation models for *fAPAR* and *LUE* were especially essential. Involving remote estimation of *fAPAR*, corresponding research has been conducted (Zhang, Zhou, & Nilsson, 2015). So in this study, we will focus on the parameter *LUE* and its quantitative algorithms. Studies indicated that the variation in foliar chlorophyll content was well correlated with temporal changes in *LUE* (Dawson, North, Plummer, & Curran, 2003; Peng et al., 2011), and it was also found that foliar chlorophyll content was a good proxy for leaf photosynthetic capacity (Croft et al., 2017). In addition, studies have shown that spectral vegetation indices (VIs) closely related to chlorophyll were used to estimate *GPP*, such as the photochemical reflectance index (PRI), which is strongly related to the photosynthetic radiation use efficiency of plant leaves (Gamon, Serrano, & Surfus, 1997; Peñuelas, Filella, & Gamon, 1995). However, its applicability at the canopy or ecosystem scales is still not well known (Ide et al., 2010; Nakaji et al., 2008; Rossini et al., 2010).

Therefore, to estimate the ecosystem *LUE* using remote sensing‐based models, we made seasonal measurements of the spectral reflectance, ecosystem CO_2_ fluxes, ecophysiological characteristics, and micrometeorological variables over a maize cropland. This study aims to estimate *LUE* for a maize canopy through exploring the relationships between the spectral VIs and photosynthetic‐efficiency or capacity‐variable canopy chlorophyll content (*CCC*_canopy_). The specific objectives were to (1) construct quantitative algorithms for *CCC*_canopy_ considering the saturation of VIs with increasing green plants; and (2) test whether the estimation models for *CCC*_canopy_ derived from field spectrometry can be effectively validated by *EC* fluxes data; and (3) ultimately assess the performance of hyperspectral remote sensing information for assessing *CCC*_canopy_. This study will provide theoretical bases for constructing ecosystem productivity models driven by full remote sensing information.

## 2 MATERIALS AND METHODS

### 2.1 Experimental site

The experimental site was located at Jinzhou Agricultural Ecosystem Research Station (41°8′53′’N, 121°12′6′’E, 23 m a.s.l.), the Institute of Atmospheric Environment, Chinese Meteorological Administration, Shenyang. It belongs to a temperate continental monsoon climate zone, with mean annual air temperature of 9°C and mean annual precipitation of 690 mm for the past 40 years. The rainfed maize is the main crop type in this area. The maize hybrid was Nong Hua 101, and it was sown in early May and harvested in late September. The maize was planted about 23 cm apart in rows and the distance of about 57 cm between rows at this experimental site. The fields are under till management and N fertilizer is around 300 kg N/ha (Han et al., 2007). The soil is a typical brown soil, which is composed of sand of 45%, silt of 40%, and clay of 15%. The pH value of the soil was 6.3, a soil organic matter content ranged from 0.6 to 0.9%, and total N was 0.069% (Han et al., 2007; Li, Zhou, & Wang, 2010; Zhang et al., 2015).

### 2.2 Field measurements

An ASD (Analytical Spectral Devices, Boulder, CO, USA) FieldSpec3 spectroradiometer with a wavelength range of 350–2500 nm was used to collect canopy spectral reflectance data biweekly from late May to late September during the whole growing season in 2011 (nine measurement campaigns). The area‐coefficient method (CMA, 1993) was used to measure leaf area index (*LAI*). A more detailed description of spectral reflectance and *LAI* measurements are given as Zhang et al. (2015).

Total *CCC*_canopy_ is an important biophysical characteristic parameter at the canopy level (Gitelson et al., 2005; Ustin et al., 1998) and is the product of *LAI* and the leaf chlorophyll content (*LCC*) (Gitelson et al., 2005). *LCC* was measured by a SPAD‐502 meter (Minolta Corporation, NJ, USA) with the same observation dates as the spectral reflectance measurements in nine campaigns. Gitelson et al. (2005) has showed that upper canopy leaves chlorophyll could be representative of the entire canopy chlorophyll. In this study, the SPAD values (M, SPAD‐502 Meter Value) at the middle‐upper positions of all green leaves for the same five observed standard plants for *LAI* were measured on each sampling date. Here, we only used the data of the third leaf from the top down, which have the most notably seasonal variations compared with other leaves, and ultimately the mean values of the five standard plants were obtained to represent the SPAD value of each sampling date. The SPAD values reflect the relative quantity of *LCC* by measuring transmission at 650 nm in the red domain and 920 nm in the infrared region (Markwell, Osterman, & Mitchell, 1995). *CCC*_canopy_ was calculated by Eqs. (1)−(3) as follows (Gitelson et al., 2005; Markwell et al., 1995):

(1) $$\text{Chlorophyll content}\left(Chl,\mu {\text{mol/m}}^{2}\right)=10^{\left(M^{0.265}\right)}$$

(2) $$\begin{array}{cc} & \text{Leaf chlorophyll content}\;\left(\mathrm{LCC},{\text{mg/m}}^{2}\right)\;=\;Chl\left(\mu {\text{mol/m}}^{2}\right)\\ & \;\times 897.01\left(\text{g/mol}\right)\times 10^{-3}\left(\text{mg}/\mu g\right) \end{array}$$

(3) $$\begin{array}{cc} & \text{Canopy chlorophyll content}(CCC_{\mathrm{canopy}},{\text{g/m}}^{2})\\ & =\mathrm{LAI}\times \mathrm{LCC}\left({\text{mg/m}}^{2}\right)\times 10^{-3}\left(\text{g/mg}\right) \end{array}$$

CO_2_ fluxes over the maize canopy at the experimental site were measured by *EC* instruments system including an open path infrared CO_2_/H_2_O gas analyzer (Li‐7500; Campbell Scientific Inc., MS, USA), a 3‐D sonic anemometer (CSAT3; Campbell Scientific Inc.) at a height of 3.5 m and an automatically stored data logger (CR5000; Campbell Scientific Inc.), as well as micrometeorological variables including air temperature and humidity (HMP45C; Vaisala, Helsinki, Finland) at heights of 2.4 m and 4.1 m, wind speed (014A/034B; Campbell Scientific Inc.), *PAR* (LI190SB; LI‐COR Inc., Lincoln, NE, USA) at the height of 3.5 m, and *SWC* (EasyAG sensors; Campbell Scientific Inc.) at depths of 10, 20, 30, and 40 cm were also measured in the 2011 growing season (Zhang & Zhou, 2014). They were installed in an undisturbed rainfed maize field occupying 43 ha with adequate fetches in all directions and uniform enough to meet requirements for *EC* measurements of carbon fluxes (Li et al., 2010).

### 2.3 Data analysis

The net ecosystem CO_2_ exchange (*NEE*) data were determined by the *EC* method as the mean covariance between fluctuations in vertical wind speed (ϖ′) and the carbon dioxide concentration (*c*′) on a half‐hourly basis (Equation (4)) (Baldocchi, 2008), and data processing and quality control procedure were conducted. To obtain complete time‐series of half‐hour CO_2_ fluxes data, the gap‐filling method of Reichstein et al. (2005) was used to fill *NEE* data. We used Equation (5) to estimate daytime ecosystem respiration (*R*_eco_) and Equation (6) to partition *NEE* into *GPP* (*GPP* = 0 during the night) and *R*_eco_. *NEE* is positive when CO_2_ is emitted from the ecosystem into the atmosphere, where *GPP* and *R*_eco_ are both positive (Reichstein et al., 2005).

(4) $$NEE=\bar{\varpi^{\prime }c^{\prime }}$$

(5) $$R_{eco}=R_{ref}\cdot e^{E_{0}\left(1/\left(T_{ref}-T_{0}\right)-1/\left(T-T_{0}\right)\right)}$$

(6) $$GPP=-NEE+R_{eco},$$

where *R*_ref_ is the ecosystem respiration at the reference temperature 10°C (mg CO_2_ m^−2^ s^−1^), *E*_0_ is the activation energy parameter (J/mol), *T* is soil temperature (°C, 0.05 m depth), *T*_0_ = 273.15 K, and a 91‐day window that can reflect the seasonal dynamics of ecosystem *R*_eco_ was applied to parameterize *R*_ref_ and *E*_0_ (Lloyd & Taylor, 1994). To match simultaneous spectral measurements over a maize canopy, the daily mean midday *GPP* values measured between 11 and 14 h were used in this study.

Eleven common chlorophyll‐related VIs were calculated in this study (Table 1). Additionally, the red edge position (REP) was used, which is particularly sensitive to green vegetation information, and was determined as the wavelength inflection point between 680 and 750 nm (i.e., the point of maximum slope) (Dawson & Curran, 1998). Four widely used VIs, *that is,* the NDVI, ratio vegetation index (RVI), wide dynamic range vegetation index (WDRVI), and 2‐band enhanced vegetation index (EVI2) (Table 1), were used to select the optimal *CCC*_canopy_ indicators using all of the possible combinations of two separate wavelengths in the range of 400–1300 nm along with 12 chlorophyll‐related VIs to explore the relationships between VIs and *CCC*_canopy_. Considering the saturation effects of VIs with an increasing *CCC*_canopy_, linear and exponential regression models were employed.

**Table 1:** Spectral vegetation indices (VIs) used in the study

Indices	Formula	References
Common chlorophyll‐related VIs		
Normalized difference vegetation index (NDVI)	(ρ_nir_–ρ_red_)/(ρ_nir_+ρ_red_)	Tucker (1979)
Enhanced vegetation index (EVI)	2.5 × (ρ_nir_–ρ_red_)/(ρ_nir_+6 × ρ_red_–7.5 × ρ_blue_+1)	Huete et al. (2002)
Ratio vegetation index (RVI)	ρ_NIR_/ρ_red_	Rouse et al. (1973)
Red edge NDVI	(ρ_750_–ρ_710_)/(ρ_750_+ρ_710_)	Gitelson and Merzlyak (1996)
Photochemical reflectance index (PRI)	(ρ_531_–ρ_570_)/(ρ_531_+ρ_570_)	Gamon et al. (1997)
Modified chlorophyll absorption ratio index (MCARI_710_)	[(ρ_750_–ρ_710_)–0.2 × (ρ_750_–ρ_550_)](ρ_750_/ρ_710_)	Wu et al. (2009)
Chlorophyll index of green (CI_green_)	(ρ_750_/ρ_550_)–1	Gitelson et al. (2005)
Chlorophyll index of red edge (CI_red edge_)	(ρ_750_/ρ_710_)–1	Gitelson et al. (2005)
MERIS terrestrial chlorophyll index (MTCI)	(ρ_753_–ρ_708_)/(ρ_708_–ρ_681_)	Dash and Curran (2004)
Canopy chlorophyll index (CCI)	D_720_/D_700_	Sims et al. (2006)
Wide dynamic range vegetation index (WDRVI)	(α×ρ_nir_–ρ_red_)/(α×ρ_nir_+ρ_red_)	Gitelson (2004)
VIs with combinations of two separate wavelengths at the range of 400–1300 nm		
Normalized difference vegetation index (NDVI)	(ρ_nir_–ρ_red_)/(ρ_nir_+ρ_red_)	Tucker (1979)
2‐band enhanced vegetation index (EVI2)	2.5[(ρ_nir_–ρ_red_)/(ρ_nir_+2.4ρ_red_+1.0)]	Jiang et al. (2008)
Ratio vegetation index (RVI)	ρ_nir_/ρ_red_	Rouse et al. (1973)
Wide dynamic range vegetation index (WDRVI)	(α×ρ_nir_–ρ_red_)/(α×ρ_nir_+ρ_red_)	Gitelson (2004)

ρ_nir*,*_ ρ_red*,*_ and ρ_swir_ are the averaged reflectance among the waveband range to match MODIS data in the near‐infrared (841–876 nm), red (620–670 nm), and shortwave infrared (SWIR1: 1628–1652 nm) wavelengths, respectively.

### 2.4 Validation of the models

According to *LUE* principles (Monteith, 1972, 1977), ecosystem *GPP* can be accurately estimated using the product of *fAPAR* and *LUE* following Equation (7):

(7) GPP=PAR×fAPAR×LUE

Considering *LUE* was closely related to ecosystem chlorophyll (Gitelson et al., 2006; Peng & Gitelson, 2011), thus, Equation (7) can be modified as the following form (Equation (8)):

(8) $$GPP=PAR\times fAPAR\times \alpha \times CCC_{\mathrm{canopy}},$$

where α is a light use coefficient for *CCC*_canopy_ per unit area, which can be parameterized by field observation data sets; *PAR* is photosynthetically active radiation; *fAPAR* is the fraction of absorbed *PAR*; and *CCC*_canopy_ is the canopy chlorophyll content.

Half‐hourly midday *GPP* between 11 and 14 h estimated and measured by an open‐path *EC* were used to effectively validate the remote estimation model for the *CCC*_canopy_ derived from hyperspectral data. All statistical analyses were performed with SPSS 17.0 software (SPSS, Chicago, IL, USA) and MATLAB R2009a software (MathWorks, Natick, MA, USA).

## 3 RESULTS AND DISCUSSION

### 3.1 Environmental variables, *LAI*, and *CCC*_canopy_

**Figure 1:**
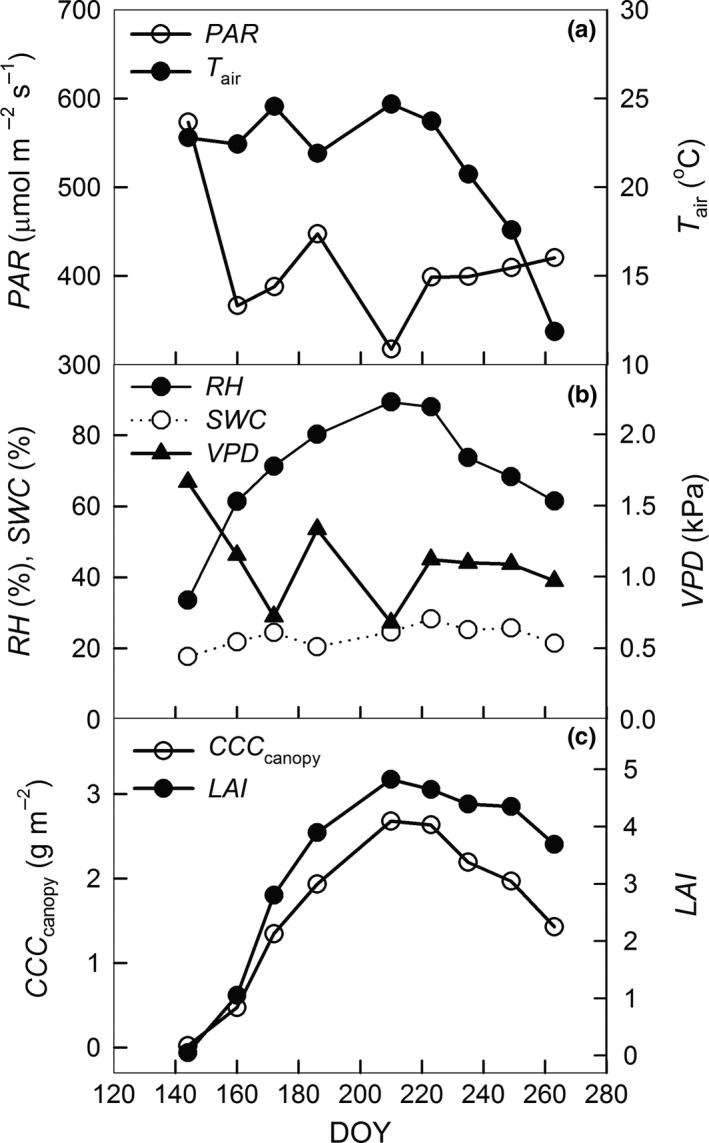
Seasonal variations of the environmental variables, canopy chlorophyll content (*CCC*_canopy_, g m^−2^), and leaf area index (*LAI*). (a) Photosynthetically active radiation (*PAR*, μmol m^−2^ s^−1^) and the mean daily temperature (*T*_air_, °C), and (b) soil water content (*SWC*, %), relative humidity (*RH*, %), and vapor press deficit (*VPD*, kPa) from micrometeorological measurements, as well as (c) *CCC*_canopy_ and *LAI*

Figure 1 shows the seasonal variations of the environmental factors, *LAI*, and *CCC*_canopy_ in the maize field. From late May to mid‐August the mean daily temperature (*T*_air_) maintained a level above 20°C and met the demands of crop growth and development (Figure 1a). *PAR* showed higher values during the early stage of the growing season and then remained at a certain level with lower values in late July (Figure 1a). Moisture factors also showed clear dynamics during the growing season (Figure 1b). Relative humidity (*RH*) showed a single‐peak seasonal trend, with values above 80% during the period from late July to early August, and reached its peak value of 89.30% on July 29 (DOY210). Vapor press deficit (*VPD*) showed large fluctuations at the early stage of the growing cycle and then gradually decreased during the late stage. Compared with *RH* and *VPD*, the seasonal variations of *SWC* were not obvious. Similar to the seasonal variation in *LAI*,* CCC*_canopy_ showed a notable single‐peak seasonal trend, which rapidly increased at the vegetative stage and gradually decreased after its peak value, occurring at the period from late July to early August (Figure 1c).

### 3.2 Relationships between chlorophyll‐related VIs and *CCC*_canopy_

**Figure 2:**
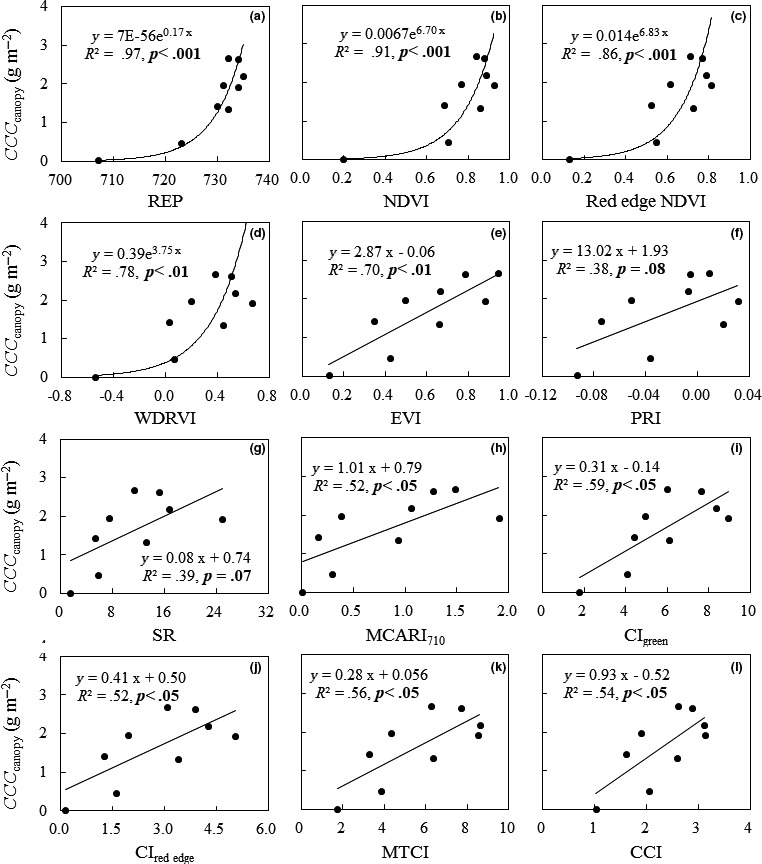
Relationships of canopy chlorophyll content (*CCC*_canopy_) with the chlorophyll‐related vegetation indices (VIs). (a) red edge position (REP), (b) normalized difference vegetation index (NDVI), (c) red edge NDVI, (d) wide dynamic range vegetation index (WDRVI), (e) enhanced vegetation index (EVI), (f) photochemical reflectance index (PRI), (g) ratio vegetation index (SR), (h) modified chlorophyll absorption ratio index (MCARI_710_), (i) chlorophyll index of green (CI_green_), (j) chlorophyll index of red edge (CI_red edge_), (k) MERIS terrestrial chlorophyll index (MTCI), and (l) canopy chlorophyll index (CCI)

Based on the relationships between VIs and *CCC*_canopy_, VIs were classified into two categories. One type of VIs had closely exponential relationships with *CCC*_canopy_, including REP, NDVI, red edge NDVI, and WDRVI, with coefficients of determination (*R* ^2^) of .97, .91, .86, and .78, respectively (Figure 2a−d). The strongest relationships exhibited between REP and *CCC*_canopy_, and NDVI and *CCC*_canopy_, although VIs gradually lost sensitivity to *CCC*_canopy_ when the latter increased above a certain level.

The other types of VIs, including EVI, PRI, SR, MCARI_710_, CI_green_, CI_red edge_, MTCI, and CCI, had obviously linear relationships with *CCC*_canopy_ (Figure 2e−l). The best linear relationship exhibited between EVI and *CCC*_canopy_, with an *R* ^2^ value of .70 (*p *<* *.01, Figure 2e). The worst relationships occurred between PRI and *CCC*_canopy_, with an *R* ^2^ value of .38 (*p *=* *.08, Figure 2f), and SR and *CCC*_canopy_, with an *R* ^2^ value of .39 (*p *=* *.074, Figure 2g); the other *R* ^2^ values were approximately .50 (Figure 2h−l). To some degree, the latter could overcome the saturation effects, but the explained variances of *CCC*_canopy_ by the linear relationships were still very limited.

Photochemical reflectance index can detect epoxidation and de‐epoxidation changes in xanthophyll relevant to heat dissipation and can be used to indicate rapid changes of the photosynthetic efficiency of photosystem II and *LUE* of plant leaves (Gamon et al., 1997; Peñuelas et al., 1995). However, at the canopy scale, the sensitivity of PRI to the variation in *CCC*_canopy_ did not perform well in this study. In addition, studies also showed that CCI could indicate changes of the chlorophyll content by the shifting of the red edge (Ide et al., 2010; Sims et al., 2006). In particular, CI_green_ [(R_NIR_/R_green_) − 1] and CI_red edge_ [(R_NIR_/R_red edge_) − 1] could effectively reflect the variation of *CCC*_canopy_ and explain more than 92% of the *Chl* variation (Gitelson et al., 2005). However, they could not be used as better proxies in this study because the effects of the canopy structure, spatial distribution of the chlorophyll content, *LAI*, and soil background decreased the reflectance signatures of *Chl* at the canopy level.

### 3.3 Relationships between VIs from the combinations of two separate wavelengths and *CCC*_canopy_

**Figure 3:**
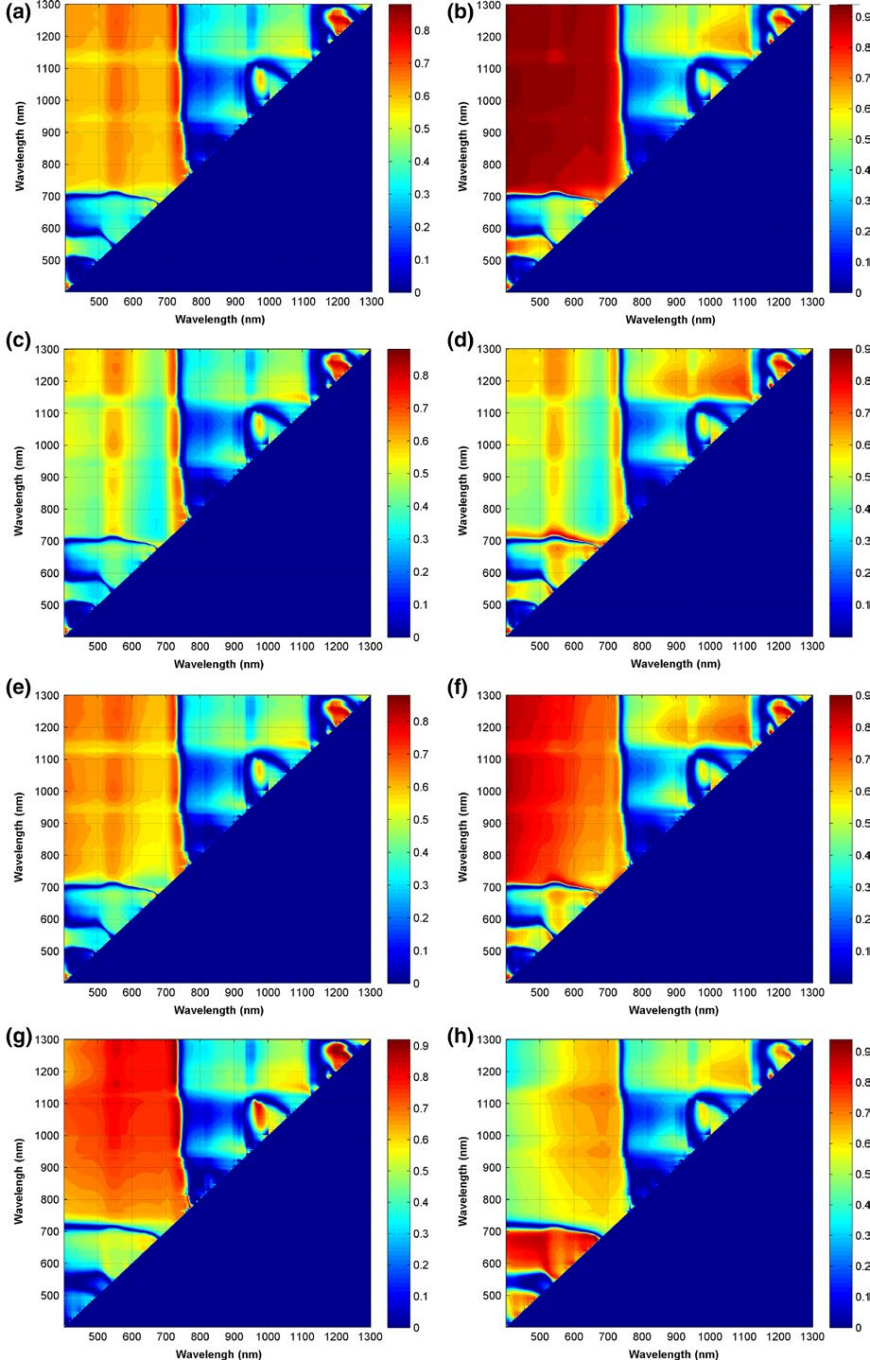
Contour maps of the coefficient of determination (*R* ^2^) for linear and exponential correlation relationships between *CCC*_canopy_ and four VIs with any combinations of two separate wavelengths in the range 400−1300 nm. (a) NDVI‐linear, (b) NDVI‐exponential, (c) RVI‐linear, (d) RVI‐exponential, (e) WDRVI‐linear, (f) WDRVI‐exponential, (g) 2‐band enhanced vegetation index (EVI2)‐linear, and (h) EVI2‐exponential. Figure 2 provides the definitions of acronyms

Figure 3 shows a contour map of *R* ^2^ between the *CCC*_canopy_ and the commonly utilized VIs, NDVI, RVI, WDRVI, and EVI2 using all of the possible combinations of two separate wavelengths in the range 400−1300 nm according to linear and exponential relationships. The *R* ^2^ value of the linear relationship NDVI [1233, 1243] versus *CCC*_canopy_ reached .89 (Figure 3a), while the *R* ^2^ value of the exponential relationship reached .95 at wavelength positions around [405, 1010], [405, 1245], and [405, 890] (Figure 3b). The exponential regression of NDVI‐*CCC*_canopy_ showed better statistical relationships between the band combinations of the visible (400–700 nm) and the near‐infrared regions (700–1300 nm). The RVI–*CCC*_canopy_ relationship was mostly not strong, with the best linear *R* ^2^ value of .89 for RVI [1233, 1243] (Figure 3c), as well as exponential *R* ^2^ values <.90 (Figure 3d). Compared with NDVI, WDRVI, to some extent, showed a similar linear relationship with *CCC*_canopy_, but it was not better than NDVI for the exponential *R* ^2^ value (Figure 3e,f).

**Figure 4:**
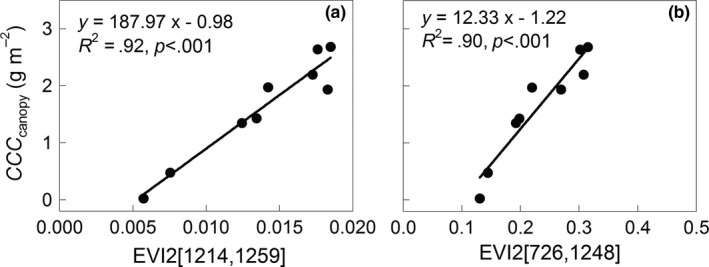
Relationships of *CCC*_canopy_ with the VIs (a) EVI2 [1214, 1259] and (b) EVI2 [726, 1248]. Figures 2 and 3 provide the definitions of acronyms.

Most of the VIs exhibited saturation effects with increasing *CCC*_canopy_, which could result in the exponential relationships between VIs and *CCC*_canopy_ being better than the linear ones (Figure 3a−f). Among the four VIs used in this study, EVI2 was the best indicator of *CCC*_canopy_ because the *R* ^2^ values of the exponential relationships between *CCC*_canopy_ and EVI2 [667, 675], *CCC*_canopy_ and EVI2 [498, 675] reached .94 and .89 (Figure 3h), respectively. Actually, good linear relationships also existed between *CCC*_canopy_ and EVI2 [1214, 1259] and *CCC*_canopy_ and EVI2 [726, 1248], with *R* ^2^ values of .92 and .90, which effectively overcame the saturation effects (Figure 4).

EVI2 proved to be suitable for accurate estimations of *CCC*_canopy_, and they were very sensitive to the *CCC*_canopy_ variations in this study. Usually, the chlorophylls have strong absorbance peaks in the red and blue regions of the spectrum. However, the blue peak is not used to estimate *Chl* because it overlaps with the absorbance of the carotenoids (Wu et al., 2009). In addition, maximal chlorophyll absorbance in the red region occurred at wavelengths from 660 to 680 nm; spectral reflectance at these wavelengths are prone to saturated light information, so they were nonsensitive, while reflectance near 550 nm in the green region and red edge region at 700 nm, where more *Chl* is required to saturate the absorption, showed greater sensitivity to a wide range of *Chl* (Wu et al., 2009). This study found that the sensitive regions to the variation in *Chl* were band combinations of the red edge region at 700−730 and 1150−1300 nm, as well as 1200 and 1250 nm (Figure 3g), which were closely related to water absorption features around 1200 nm. Although linear and exponential relationships between *CCC*_canopy_ and four VIs using any combinations of two separate wavelengths in the range 400−1300 nm were constructed based on only statistical relationships, from which the possible sensitive spectral features or spectral ranges to the variations of *CCC*_canopy_ were clearly presented. Certainly, more investigations are necessary to further validate their effectiveness and feasibilities for satellite data at broader spatial scales.

### 3.4 Validation of the hyperspectral remote estimation of *CCC*_canopy_

Crop *GPP* was strongly related to *CCC*_canopy_, *Chl* per unit area to a large extent determined crop productivity, net photosynthesis, and light absorbance (Peng & Gitelson, 2011). Moreover, long‐ or medium‐term changes in *CCC*_canopy_ were closely related to crop phenology, canopy stresses, and photosynthetic capacity, thus it was an important physiological variable that strongly related with productivity at the community level (Gitelson et al., 2005, 2006; Ustin et al., 1998).

Half‐hourly midday *GPP* between 11 and 14 h estimated and measured by an open‐path *EC*, through in combination with the algorithms of *fAPAR* calibrated by green *LAI* (*fAPAR*_green_) (Zhang et al., 2015) and *PAR* from meteorological observations were utilized to validate the remote estimation models for the *CCC*_canopy_. Studies derived from the same field measurements, including spectral measurements and crop canopy data from *fAPAR* observations, showed that NDVI was a good predictor of *fAPAR*_green_ as Equation (9) (Zhang et al., 2015):

(9) $$fAPAR_{\mathrm{green}}=1.235\times \text{NDVI}-0.211\left(R^{2}=.90,P<.001\right)$$

Here we established *CCC*_canopy_ algorithms based on hyperspectral data including NDVI and REP (Figure 2a,b), EVI2 [1214, 1259] and EVI2 [726, 1248] (Figure 4) derived from the optimal band combinations as Equations (10)–(13):

(10) $$CCC_{\mathrm{canopy}}=0.0067\times e^{\left(6.6986\times \mathrm{NDVI}\right)}\left(R^{2}=.91,\;P<.001\right)$$

(11) $$CCC_{\mathrm{canopy}}=6.9002\times 10^{-56}\times e^{\left(0.1743\times \mathrm{REP}\right)}\left(R^{2}=.97,\;P<.001\right)$$

(12) $$CCC_{\mathrm{canopy}}=187.97\times \text{EVI2}\;\left[1214,1259\right]-0.98\left(R^{2}=.92,\;P<.001\right)$$

(13) $$CCC_{\mathrm{canopy}}=12.33\times \text{EVI2}\;\left[726,1248\right]-1.22\left(R^{2}=.90,\;P<.001\right)$$

**Figure 5:**
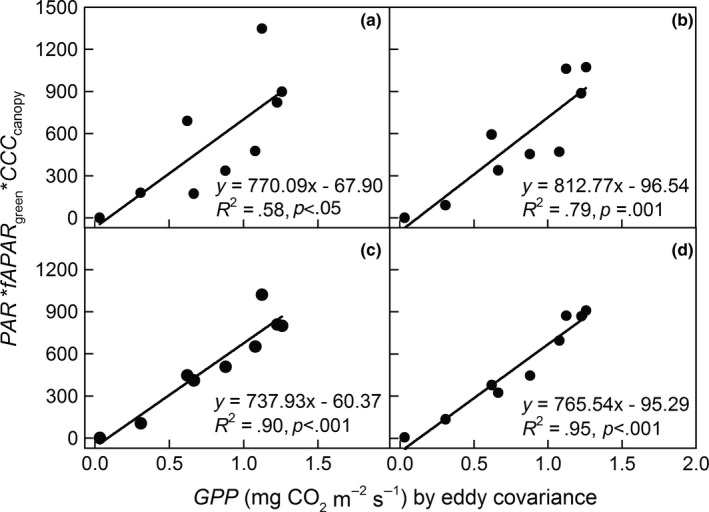
Comparisons of the estimated *PAR* (photosynthetically active radiation) * *fAPAR*_green_ (the fraction of absorbed photosynthetically active radiation calibrated by green *LAI*) * *CCC*_canopy_ using the VIs and gross primary productivity (*GPP*) derived from the eddy covariance observations. (a) *CCC*_canopy_ estimated by NDVI, (b) *CCC*_canopy_ estimated by REP, (c) *CCC*_canopy_ estimated by EVI2 [1214, 1259], and (d) *CCC*_canopy_ estimated by EVI2 [726, 1248]. Figures 2 and 3 provide the definitions of acronyms.

Figure 5 shows that the estimated *GPP* values driven by *LUE* principles and the measured *GPP* derived from *EC* used to validate were closely related, and satisfactory linear relationships were obtained (*R* ^2^ = .58−.95, Figure 5). Among Equations (10)–(13), EVI2 [726, 1248] via Equation (13) was the best algorithm for *CCC*_canopy_ estimation (*R* ^2^ =. 95, *p *<* *.001, Figure 5d). According to Equation (8), when obtaining the coefficient α for *CCC*_canopy_ per unit area, which reflect different radiation use abilities of different monitoring indicators for *CCC*_canopy_ per gram and per unit area in maize ecosystems, ecosystem *GPP* in maize could be estimated based on *LUE* model (Equation (8)) and remote sensing data (Equations (9)–(13)). This study further demonstrated that based on *LUE* principles, a *CCC*_canopy_ algorithm derived from field spectrometry measurements through in combination with an algorithm of *fAPAR*_green_ and *PAR* from meteorological observations could be used to estimate *GPP* in maize agricultural ecosystems.

## 4 CONCLUSIONS

This study investigated remote estimation of *LUE* through estimating *CCC*_canopy_ based on field measurements of spectral reflectance, *Chl*,* LAI*, and ecosystem CO_2_ fluxes as well as micrometeorological factors conducted during the entire growing season for a maize canopy. Among the common chlorophyll‐related VIs, REP and NDVI had better exponential relationships with *CCC*_canopy_, although there existed a certain saturation effect with increasing *CCC*_canopy_; to some degree, EVI, PRI, SR and so on, could overcome the saturation effects, the explained variances of *CCC*_canopy_ by the linear relationships were still very limited. Thus to select the most sensitive spectral information, when estimating *CCC*_canopy_ using all of the possible combinations of two separate wavelengths in the range of 400–1300 nm, EVI2 [1214, 1259] and EVI2 [726, 1248] were proved to be the best indicators of *CCC*_canopy_. This study demonstrated that hyperspectral remote sensing information could effectively monitor the seasonal variations of *CCC*_canopy_. Although more researches are needed to validate the performance of spectral features for estimating *CCC*_canopy_, we believe that the selected sensitive indicating spectral information will be attractive for actual applications of satellite data at broader temporal and spatial scales.

This study further demonstrated that based on *LUE* principles, a *CCC*_canopy_ algorithm derived from field spectrometry measurements through in combination with an algorithm of *fAPAR*_green_ and *PAR* from meteorological observations could be used to monitor midday *GPP* in maize agricultural ecosystems. We optimized the parameterization of *LUE* using field spectrometry observation data sets, developed an ecophysiological based *LUE* model, and it showed a good performance. However, considering limited observations in this study, more studies in the future are still necessary to validate this new conceptual model for monitoring vegetation *GPP* based on the combination of *LUE* models with plant physiological properties.

## CONFLICT OF INTEREST

None declared.
